# Extracellular Vesicles in Brain Tumors and Neurodegenerative Diseases

**DOI:** 10.3389/fnmol.2017.00276

**Published:** 2017-08-31

**Authors:** Federica Ciregia, Andrea Urbani, Giuseppe Palmisano

**Affiliations:** ^1^Department of Pharmacy, University of Pisa Pisa, Italy; ^2^Department of Clinical and Experimental Medicine, SOD Endocrinology and Metabolism of Organ and Cell Transplants, University of Pisa Pisa, Italy; ^3^Istituto di Biochimica e Biochimica Clinica, Università Cattolica Rome, Italy; ^4^Proteomics and Metabonomics Unit, IRCCS-Fondazione Santa Lucia Rome, Italy; ^5^GlycoProteomics Laboratory, Department of Parasitology, Institute of Biomedical Sciences, University of Sao Paulo Sao Paulo, Brazil

**Keywords:** exosomes, central nervous system, brain tumors, neurodegenerative diseases, biomarkers

## Abstract

Extracellular vesicles (EVs) can be classified into apoptotic bodies, microvesicles (MVs), and exosomes, based on their origin or size. Exosomes are the smallest and best characterized vesicles which derived from the endosomal system. These vesicles are released from many different cell types including neuronal cells and their functions in the nervous system are investigated. They have been proposed as novel means for intercellular communication, which takes part not only to the normal neuronal physiology but also to the transmission of pathogenic proteins. Indeed, exosomes are fundamental to assemble and transport proteins during development, but they can also transfer neurotoxic misfolded proteins in pathogenesis. The present review will focus on their roles in neurological diseases, specifically brain tumors, such as glioblastoma (GBM), neuroblastoma (NB), medulloblastoma (MB), and metastatic brain tumors and chronic neurodegenerative diseases, such as Alzheimer, Parkinson, multiple sclerosis (MS), amyotrophic lateral sclerosis (ALS), Huntington, and Prion diseseases highlighting their involvement in spreading neurotoxicity, in therapeutics, and in pathogenesis.

## Introduction

Extracellular vesicles (EVs) comprise a wide variety of membrane-limited vesicles released from cells. They can be broadly classified into three subclasses, based on their origin or size: apoptotic bodies, microvesicles (MVs), and exosomes (Crescitelli et al., 2013; Abels and Breakefield, 2016).

Apoptotic bodies represent the largest EVs with size ranging from 1,000 to 5,000 nm; the name is due to their origin, since they are released as blebs from cells undergoing programmed death cell. MVs vary in size from 100 to 1,000 nm and are produced by external budding from the plasma membrane. Finally, exosomes are the smallest vesicles (30–100 nm) and derived from the endosomal system. Indeed, they do not originate from plasma membrane, but they are derived from internal budding of vesicles in the lumen of early endosome (Raposo and Stoorvogel, 2013; Yáñez-Mó et al., 2015; Ban et al., 2016; Kalamvoki and Deschamps, 2016). Thereby, resulting in the formation of intracellular multivesicular bodies, which successively fuse with the plasma membrane, and liberate exosomes to the extracellular medium (Pan and Johnstone, 1983). Exosomes are the best characterized EVs, that were firstly described in the early ‘80s as vesicles containing 5′-nucleotidase activity (Trams et al., 1981).

Initially, exosomes were thought to be a cellular mechanism for discarding unwanted materials from cells (Pan and Johnstone, 1983). Nowadays, there is an increasing interest in studying exosomes as specifically-secreted vesicles enabling communication among cells which is involved not only in normal physiological process but also in pathological diffusion (Vlassov et al., 2012; Abels and Breakefield, 2016). Exosomes can exchange membrane proteins and cytosol between cells and activate signaling pathways in recipient cells (Kalluri, 2016), and the role of the vesicles is defined by exosomes composition. Exosomes contain lipids, proteins, DNA, miRNA, mRNA, long non-coding RNA, and genetic materials from viruses or prions, depending on cellular origin and putative target function.

They are released from many different cell types, including neuronal cells (Fauré et al., 2006) and their functions in the nervous system are currently under active investigation. Exosomes are involved in the function of nervous system, including the regulation of synaptic communication and strength, and nerve regeneration (Antonucci et al., 2012; Fröhlich et al., 2014; Park et al., 2015). They have been proposed as a novel means for intercellular communication which takes part not only to the normal neuronal physiology but also to the transmission of pathogenic proteins (Février et al., 2005; Smalheiser, 2007; Park et al., 2015). Indeed, they are fundamental for the packaging and transport of both proteins during development and neurotoxic proteins during pathogenesis (Park et al., 2015).

This review will focus on the roles these vesicles play in neurological diseases and discuss their involvement in spreading neurotoxicity, in therapeutics, and in pathogenesis for these diseases.

## Exosomes in brain cancers

The clinical complexity of many brain tumors is related to the peculiar microenvironment of the central nervous system (CNS) and to its intra/inter-cellular interactions; this contributes to make brain cancer a difficult medical challenge (D'Asti et al., 2012, 2016). Therefore, exosomes are a form of cellular interaction which can offer a tool for cancer progression. Indeed, tumor cells release diffusible factors through exosomes, which contribute to tumor. The secretion of exosomes by malignant cells can result in signaling process that leads to abnormal cell growth and can contribute to the hypoxic environment which is typical of tumors (Kucharzewska et al., 2013). Therefore, progress in isolating and identifying these factors can contribute to cancer therapeutics development. Here we describe the role of exosomes in the progression of different types of brain cancer.

### Glioblastoma

Glioblastoma (GBM) is the most aggressive among tumors of glial origin with a median patient survival of 12–15 months (Dolecek et al., 2012) and exhibits aggressive infiltrative growth patterns. GBM is an extremely heterogeneous cancer, with variety of subtypes which contribute to the complexity in its study. It is composed by cancer stem cells, tumor cells and non-neoplastic parenchymal cells, comprising vascular cells, microglia, and peripheral immune cells (Wu et al., 2010; Setti et al., 2015). The communication among these cells via the secretion of exosomes has a pivotal role in the course of pathology. Indeed, glioma cells do not exit the CNS (Mourad et al., 2005), therefore exosomes and/or other types of MVs may influence processes distant from the tumor site. Graner et al. suggested that these processes could include immune responses. With a first study in 2007, they identified exosomes in the spent media from human and murine glioma by differential centrifugation (Graner et al., 2007).

In particular, Graner et al showed that exosomes released from D54MG and SMA560 human brain tumor cells contains heat shock proteins HSP27, 60, 70, and 90 (Graner et al., 2007). Moreover, mice vaccinated with a chaperone-rich cell lysate isolated from tumor tissues had an increased splenocyte reactivity and reduced tumor growth (Graner et al., 2009). Kore el al. analyzed by mass spectrometry the exosomes secreted by U373 human brain cells and identified Alpha-crystallin B chain (CRYAB), also known as HspB5 (Kore and Abraham, 2014). In this study, treatment with pro-inflammatory cytokines, such as TNF-α and IL-1β increased the release of HspB5, suggesting a role in tumor progression by conferring resistance to cell death. It should be noted that no functional validation was reported in this study. The apparent discrepancy between the two studies could be related to the cell line used and the chaperones investigated which are involved in different pathophysiological mechanisms.

The hint that GBM exosomes contain immunomodulatory agents was further pursued. Harshyne and collaborators observed that GBM cell lines secrete exosomes with high immunogenic potential in humans and mice (Harshyne et al., 2015, 2016). They found that serum from GBM patients with high levels of exosome-reactive antibodies, had also high numbers of exosomes which drive monocyte polarization toward M2 *in vitro* supporting tumor growth, while this was not observed in exosomes from normal sera (Harshyne et al., 2016).

Since exosomes are present in almost all human body fluids, (i.e., saliva, blood plasma, cerebrospinal fluid (CSF), urine) they are particularly promising as reservoirs of diagnostic and prognostic biomarkers. Biomarkers could be clinically meaningful in allowing the early detection of the tumor and when biopsy results are inconclusive. In glioma, elevated miR-221 expression is a biomarker for glioma. In a study of 2015 EVs were isolated from GBM cell lines, plasma and CSF of GBM patients (Akers et al., 2015). The aim was to investigate the relative distribution of miRNA within subpopulations of EVs which were fractionated using differential centrifugation. miR-21, togheter with other GBM-pertinent miRNAs, are highly enriched in EVs derived from CSF of GBM patients while they are not detectable in EV depleted CSF, confirming previous results (Akers et al., 2013). In particular, Akers et al. described that miRNAs were enriched in the exosome fraction from CSF; this data suggested that CSF-EV based diagnostic involving the miRNAs should be focused on the exosome fraction (Akers et al., 2015). On the other hand, they did not find such unambiguous results with plasma; it is likely that the complex pattern reflected the well-recognized complexity of plasma.

Plasma was the fluid analyzed in GBM patients by Muller et al. (2015). They affirmed that changes in overall exosomal proteins and mRNA levels could act as markers of immunological and clinical responses in GBM patients which have received antitumor vaccines (Muller et al., 2015). The miRNA expression was also investigated in serum exosomes of GBM patients. In this hypothesis-generating study, the authors proposed that miR-320, miR-574-3p, and RNU6-1 could serve as potential diagnostic biomarkers (Manterola et al., 2014).

Recently, a new exosomal RNA analysis platform: iMER (immuno-magnetic exosome RNA analysis) has been developed. It is based on the enrichment of EGFR/EGFRvIII exosomes, and quantifying their mRNA contents in real time (Shao et al., 2015). The EGFRvIII mutant is specific, characterizing a clinical subtype of glioma; moreover, EGFRvIII was not found in serum exosomes from normal control individuals (Skog et al., 2008). With their study Shao et al. (2015) illustrated how exosomal mRNA profiles could be correlated to treatment response in GBM patients. Then, exosomal mRNA and protein cargo appeared to be informative for monitoring responses to glioma immunotherapy (Muller et al., 2015), but the use of proteomics could give a more complete instrument for investigating GBM (Shao et al., 2012). In this perspective, Mallawaaratchy and colleagues have recently analyzed by quantitative high-resolution mass spectrometry EVs secreted by GBM cell lines (Mallawaaratchy et al., 2017). They found that gene levels corresponding to five EV proteins involved in cancer invasion (annexin A1, actin-related protein 3, integrin-β1, insulin-like growth factor 2 receptor and Alix) were significantly higher in GBM lesions compared to normal brain. Moreover, they showed that Cavitron Ultrasonic Surgical Aspirator (CUSA) washings are a novel source of brain tumor-derived EVs. This could foster the translation to clinical relevant blood-based biomarkers for GBM.

### Neuroblastoma

Neuroblastoma (NB) is a neoplasm of the sympathetic nervous system, and it is the second most common extracranial malignant tumor of childhood, that poses a significant risk of death (Park et al., 2010). Since it is a complex disease, factors, such as the age at diagnosis, and the features of the tumor will guide the tumor prognosis toward a spontaneous regression or metastatic progression becoming refractory to therapy, as assessed by Cheung and Dyer (2013). Despite the recent improvements in treating this disease, new therapeutics are needed and can derive from enhanced understanding of the NB biology. One area of investigation are exosomes. The extracellular trafficking of complex biological messages would offer a key to understand NB and to find novel therapeutic targets.

To date, only few studies were devoted to the involvement of EVs in NB. In 2015 a study by Haug et al. showed for the first time that MYCN-amplified NB cell lines secrete exosome-like particles containing miRNAs (Haug et al., 2015). MYCN is a proto-oncogene which is related in high-risk NB. The vesicles were shown to be taken-up by recipient cells, releasing miRNAs with potential roles in cancer progression.

Exosomic miRNAs have also been studied by Challagundla and collaborators. They hypothesized that tumor-associated macrophages affect NB resistance to chemotherapy by exchanging of exosomal miRNAs (Challagundla et al., 2015). co-culture experiments were performed by this group in order to evaluate the transfer through exosomes of miR-155 from human monocytes to NB cells and miR-21 from NB cells to human monocytes.

They showed a new role of exosomal miR-155 and miR-21 in the communication between human monocytes and NB cells in the resistance to chemotherapy, by a unique exosomal miR-21/TLR8-NF-κB/exosomic miR-155/TERF1 signaling pathway (Challagundla et al., 2015). Indeed, the “educational” process caused by NB on human monocytes by the secretion of exosomal miR-21 results in the up-regulation of miR-155 in NB cells dependent by TLR8 and NF-κB. Once in NB cells, miR-155 directly target TERF1, an inhibitor of telomerase whose activity correlates with drug resistance and poor outcome in many malignancies (Ohali et al., 2006; Smith et al., 2009), and telomerase activity. Moreover, they identified exosomes within the tumor microenvironment as an important molecular target to strengthen drug sensitivity.

The first proteomic analysis of exosomes derived from human NB cell lines was by Marimpietri et al. (2013) which employed two-dimensional microchromatography coupled with an ion trap mass spectrometer. They identified 390 proteins and the 20% of these exosomic proteins derived from NB represent a “signature” of cells of neuroblastic origin. Among the exosomal proteins, the ones with highest scores were fibronectin and clathrin. The first contributes to establish the inflammatory microenvironment which supports tumor growth pivotal role, while clathrin is involved in the formation of vesicles. In addition, they found many proteins that regulate the tumor microenvironment promoting tumor progression (Marimpietri et al., 2013).

### Medulloblastoma

Medulloblastoma (MB) is the most common malignant brain cancer in childhood, consisting of around 20% of all pediatric brain tumors with the tendency to disseminate at an early stage (Dhall, 2009; Coluccia et al., 2016). MB includes clinically and molecularly different cancer subtypes which include the most common malignant childhood brain tumors which have distinct cellular origins (Gibson et al., 2010). This clinical heterogeneity within MB patients makes response to therapy and overall survival markedly variable, hindering the development of much needed new therapies. Indeed, the current therapies involve surgery followed by radiation therapy and chemotherapy, but in childhood craniospinal irradiation is rarely used because it entails the risk of long-term neurocognitive deficits. Thus, to gain new information on the molecular mechanism of MB, especially with regard to their proliferative behavior, and their abilities to divert immune responses, can help in developing new treatment strategies to improve cure rates.

The first which investigated the potential role of exosomes in MB pathogenesis, were Epple and collaborators. They analyzed exosomes in MB cell line with proteomic and biochemical analyses, and characterized serum exosomes (Epple et al., 2012). Their results indicated rather high percentages of nuclear proteins, but also the presence of proteins with binding properties typical of nucleic acid and involved in transcriptional regulation. They confirmed the presence of the canonical HSP (Graner et al., 2009), moreover, they discovered signaling molecules/tumor antigens GPNMB and ERBB2 (Her2/neu), the tumor transcription factor HNF4A, and common exosomal molecules, like tetraspanin CD9 (Epple et al., 2012). Their work with patient serum exosomes implied that GPNMB and/or ERBB2 can be exosomal markers specific of tumor. Additionally, they suggested a role for HNF4A in MB biology. HNF4A could be a tumor suppressor (Ning et al., 2010); indeed, they sought to inhibit it with MEDICA 16, a known drug inhibitor, observing an increase in cell proliferation. Thus, this work showed that tumor exosomes may have a role in growth through the HNF4 transcription factor.

A more recent study identified, with a proteomic approach, a set of proteins carried by vesicles originating from MB cell lines cultured both in standard conditions of adhesion and as spheres (MBS) (Bisaro et al., 2015). Comparing their proteomic analysis with earlier proteomic studies on MB and exosomes (Graner et al., 2009; Epple et al., 2012), Bisaro et al. found that many proteins are shared, such as G3P, ANXA2, TRFE, GRP78, ACTB, HBB, CH60, and HSP90. Other proteins are different, but are part of the same functional classes (e.g., zinc finger proteins, tubulin polymerization promoting protein family member, histone proteins) (Bisaro et al., 2015). Intriguinly, iron carrier proteins (i.e., Serotransferrin and Hemopexin) were just found in vesicles emitted by the cancer stem cells-enriched tumor cell population. Iron depletion causes cell arrest in G1 phase leading to apoptosis (Steegmann-Olmedillas, 2011). Bisaro et al. found that the use of iron chelators in MBS culture caused the decrease in the size and number of spheres and determined also a reduction in stem cell populations capable to initiate the formation of *in vitro* spheres. Therefore, this study suggested the possibility to use iron chelators in antitumoral therapy.

These results are encouraging for future research efforts to understand the effect of exosomal proteins useful for the development of new chemotherapies.

### Metastatic brain tumors

Metastatic brain tumors represent a complex question due to the number of extracranial malignancies that can metastasise to the CNS. Brain metastasis commonly occurs in patients with breast and lung cancers. Approximately, 60% of all metastatic brain tumors are imputable to non-small cell lung cancer, and brain metastasis also affects up to 30% of breast cancer patients (Palmieri et al., 2006; Garnier et al., 2013).

It is reasonable to predict that exosomes can be implicated in pro-metastatic events: molecules present on tumor-derived exosomes can be horizontally transferred to recipient cells of specific organs. To date, only few observations has been translated into this field of study.

Fong et al. demonstrated that miR-122 is highly secreted by breast cancer cells and can promote metastasis (Wu et al., 2012; Fong et al., 2015). MiRNA can be secreted into the extracellular environment through exosomes and transferred to neighboring or distant cells to modulate cell function. To study the effect of miR-122, they focused on lung fibroblasts, brain astrocytes, and neurons that are abundantly present in the pre-metastatic sites of breast cancer. They affirmed that cancer-secreted miR-122 can be transferred to normal cells in the pre-metastatic niches, thereby suppressing glucose utilization in these cells to accommodate the massive energy needs of cancer cells during metastatic growth (Fong et al., 2015). Moreover, *in vivo* inhibition of miR-122 restored glucose uptake in distant organs, such as brain, decreasing the incidence of metastasis.

Tominaga et al. focus their attention on a different miRNA. Their work suggests that cancer-derived EVs promote the breakdown of blood–brain barrier via delivery of proteins and miRNAs. Importantly, miR-181c seems to have an important role in malignancy. miR-181c leads the down-regulation of its target gene, PDPK1. This results in the breakdown of blood–brain barrier by activated cofilin which disassembles actin filaments. It is noteworthy to quote that the authors observed in breast cancer patients with brain metastasis a raised level of circulating miR-181c, and cancer-derived EVs had a more evident tendency to accumulate in the brain respect to those from the parental cell line (Tominaga et al., 2015).

For a better understanding of the role of exosomes to foster metastatic brain tumors, it is interesting the study by Hoshino and collaborators published in 2015 (Hoshino et al., 2015). Earlier, the same group showed that exosomes are one of the elements derived from tumor which fosters inflammation, metastasis and recruitment of bone marrow progenitor cells during the conditioning of the secondary tumor niche (Peinado et al., 2012). Thereafter, they characterized the exosomal proteome of several tumor models (e.g., colorectal, breast, gastric and pancreatic cancers), which show a propensity to metastasize, also in brain. Analyzing the bio-distribution of tumor-secreted exosomes they found that exosomal integrins promote adhesion with target cells in a tissue-specific way and also elicit signaling pathways and inflammatory responses in target cells determing the growth of metastatic cells (Hoshino et al., 2015).

An extensive work by Zhang et al. revealed a complex cross-talk among metastatic tumor cells and their brain tumor microenvironment which promotes the brain metastases (Zhang et al., 2015). Zhang and colleagues demonstrated that exosomal miR-19a, derived from astrocyte, determines the reversible downregulation of the tumor suppressor PTEN in cancer cells, thereby increasing CCL2 secretion and recruitment of myeloid cell to support brain metastasis. In addition, by analyzing the protein levels, the authors confirmed the significant down-regulation of PTEN and higher levels of CCL2 in brain metastases respect to primary breast cancers. Notably, the brain metastatic ability of cancer cells was not modified by the knock-out of PTEN expression and PTEN expression upon cell culture was retrieved by brain metastasis. Thus, they hypothesized that PTEN down-regulation was induced on tumor cells from the brain tumor microenvironment. By co-culture experiments, the authors identified astrocytes as the cells behind this reversible non-genetic PTEN loss in brain metastases (Zhang et al., 2015).

Further researches in this field could yield novel insights into the molecular mechanisms of metastasis and prime the development of advanced therapeutic strategies to prevent the formation of brain metastasis.

## Exosomes in chronic neurodegenerative diseases

Within neurological disorders, the contribution of exosomes is most studied in neurodegenerative diseases, especially in Alzheimer's and Parkinson's diseases. Neurodegenerative disorders are characterized by a progressive loss of function and/or structure of neurons, including death of neurons. Exosomes may have a neuro-protective or neuro-toxic role in these pathological processes of CNS. Indeed, vesicles can mediate removal of toxic proteins or transfer neuro-protective exosomal molecules. On the other hand, exosomes can spread potentially toxic molecules into the recipient neural cells. A number of studies focused on their role in disease propagation and pathology and their usefulness as a diagnostic tool.

### Alzheimer's disease

Alzheimer's disease affects around 30 million patients worldwide. The majority of patients are sporadic and of late onset (around and over 65 years old) while only 5% of patients have early onset and related to genetic factors (Blennow et al., 2006; Burns and Iliffe, 2009; Querfurth and LaFerla, 2010; Hung et al., 2016). Dementia is the major manifestation of the disease, starting initially with the loss of short term memory (Hung et al., 2016). The past 30 years of Alzheimer's disease research have led to the certain proof that accumulation of abnormally folded amyloid β (Aβ) and tau proteins (hyper-phosphorylated tau; p-tau) in amyloid plaques and neuronal tangles, respectively, are causally connected to the typical neurodegenerative process in patients' brains (Karran et al., 2011; De Strooper and Karran, 2016; Scheltens et al., 2016).

By now, some notions about Alzheimer's disease hold true (Hung et al., 2016): (i) neurons are the primary cells dying, by apoptosis, in brains; (ii) plaques conduct to develop death enzymes in neurons, such as caspases, in particular, caspase 6 is an apoptotic cysteine protease which induces disease progression promoting neurodegeneration (Vaidya et al., 2011); (iii) brain derived neurotrophic factor (BDNF) has several roles in synaptic plasticity and neuronal survival and it is downregulated in Alzheimer's disease (Janel et al., 2017); (iv) in Alzheimer patients, function and levels of adhesion molecules viable for synapses are affected, e.g., synaptic cell adhesion molecules which are glycoproteins of cell surface (Leshchyns'ka and Sytnyk, 2016) (v) Alzheimer brains are characterized by a decrease of neurotransmitters, primary acetylcholine, but also catecholamine, glutamate, and serotonin (Selkoe and Schenk, 2003; Hung et al., 2016).

The hint that exosomes may take part to the spreading of pathology has been substantiated since 2006 by Rajendran and coworkers. The Aβ peptide is derived from a sequential processing which involves cleavages of the amyloid precursor protein (APP). β-secretase cleaves APP generating secreted APP and the C-terminal fragment (C99) with 99 amino acids which is tied to the membrane. Then γ-secretase cleaves C99 producing Aβ which is secreted. Aβ varies from 38 to 42 amino acids since γ-secretase catalyzes an imprecise cleavage (Selkoe and Schenk, 2003; Kandalepas and Vassar, 2012). They found that β-cleavage takes place in a specific subset of endosome and that a 1% of Aβ is secreted into the extracellular medium tied to exosomes. In addition, their finding that proteins associated with exosome (e.g., flotillins, Alix) are enriched in the amyloid plaques, suggested that exosome-associated Aβ may be involved formation of plaques (Rajendran et al., 2006). In this perspective exosomes have been proposed as the “Trojan horses of neurodegeneration” (Rajendran et al., 2006; Ghidoni et al., 2008). The release of Aβ by exosomes, although little, may be involved in the slow progression of the disease, similarly to prion proteins which mediate their intercellular transfer via exosomes (Fevrier et al., 2004).

The role of exosomes in Alzheimer's disease was further explored and a novel secretion pathway of APP metabolites, including C-terminal fragments (CTFs) of APP, mediated by exosome vesicles, has been demonstrated (Vingtdeux et al., 2007; Sharples et al., 2008). Perez-Gonzalez et al., isolating vesicles from the brains of transgenic mice over expressing human APP (Tg2576), noticed raised levels of full length APP, CTFs–APP, and Aβ compared to wild type mice (Perez-Gonzalez et al., 2012).

Furthermore, members of the secretase (family of proteases which catalyzed the amyloidogenic cleavage of APP) were identified associated with exosomes, corroborating the fact that cleavage of APP may occur within these vesicles (Sharples et al., 2008). Thus, Sharples et al. proposed that exosomes could be considered as a diagnostic and therapeutic target (Sharples et al., 2008).

In 1995, amyloid β-protein was characterized by its strict binding to GM1 ganglioside (Yanagisawa et al., 1995). The same group subsequently observed that the treatment with chloroquine, an agent that prevents vesicles trafficking, significantly accelerated the release of exosomes-associated GM1 (Yuyama et al., 2008). Remarkably, GM1 on exosomal surface was sufficient for inducing fibrillogenesis of exogenously added Aβ. Therefore, their results suggested that the release of exosomal GM1 is another pathway of deposition in the Alzheimer's disease brain, induced by endocytic pathway abnormality.

Some authors proposed a neuro-toxic role for exosomes in Alzheimer's disease with a study on primary cultured astrocytes, Wang et al. reported a new mechanism of apoptosis induction by PAR-4/ceramide-enriched exosomes, which can contribute to Alzheimer's disease (Wang et al., 2012). Indeed, in their experiments, apoptosis was thwarted by shRNA-mediated down-regulation of PAR-4, a protein which sensitize cells to the sphingolipid ceramide. Moreover, they showed that amyloid peptide induced the secretion of PAR-4 /C18 ceramide-enriched exosomes. While apoptosis was not noticed in astrocytes with deficient neutral sphingomyelinase 2 (nSMase2), suggesting that ceramide generated by nSMase2 is important for apoptosis induced by amyloid (Wang et al., 2012). The group carried on this field of analysis, and identified nSMase2 as a potential drug target in Alzheimer's disease by preventing exosome secretion (Dinkins et al., 2014). They showed that pharmacological inhibition of nSMase2 reduces amyloid burden and plaque load in Alzheimer's disease brains *in vivo*, indicating that this drug intervention may offer new treatment options for Alzheimer's disease (Dinkins et al., 2014). Moreover, they tried to administer ceramide to increase serum anti-ceramide antibodies in 5XFAD mice and suggested that this could decrease Aβ levels and serum exosome levels (Dinkins et al., 2015). However, ceramide-treated mice had more serum exosomes, suggesting that systemic anti-ceramide IgG and exosome levels are associated with the increase of plaque number (Dinkins et al., 2015).

The role of EVs in propagation of disease was supported by the work by Agosta et al. which examined EVs in CSF from patients and controls. They found higher levels of myeloid vesicles in Alzheimer's disease patients. Moreover, levels of these EVs were related with atrophy of hippocampus and the activation of microglia, resulting in an increased EVs release, was connected with the neuronal loss extent (Agosta et al., 2014).

The neuro-toxic role of EVs was further supported by Joshi et al. They found an high release of MVs in CSF of Alzheimer's disease patients; besides, EVs can drive neurodegeneration. Authors suggested that vesicles released by microglia, through their lipid composition (e.g., sphingomyelin, cholesterol, ceramide, flotillin-2, GM1, and GM3 gangliosides), may promote formation of neurotoxic soluble forms of Abeta starting from internalization of extracellular insoluble aggregates (Joshi et al., 2014).

There are also works that suggest how exosomes may have a protective role in Alzheimer's disease.

For example, cystatin C was found secreted by mouse primary neurons in association with exosomes by means of immunoproteomic analysis using SELDI-TOF MS, and cystatin C has a neuroprotective role in Alzheimer's disease (Sundelöf et al., 2008; Ghidoni et al., 2011). Moreover, low serum cystatin C levels seem to precede clinical sporadic Alzheimer's disease (Sundelöf et al., 2008).

An and collaborators still sustained the protective role of exosomes. Indeed, their study provided data that exosomes from N2a cells or human CSF may cancel the synaptic-plasticity-disrupting activity of both synthetic and Alzheimer's disease brain-derived Aβ (An et al., 2013). It has also been proposed that the decrease in β-amyloid would be mediated by delivering exosomes loaded with siRNA targeted to BACE1, a therapeutic target in Alzheimer's disease (Alvarez-Erviti et al., 2011a).

Yuyama and co-workers devoted many studies to exosomes and Aβ. They identified novel roles for neuronal exosomes on the extracellular Aβ. In particular, Yuyama et al. proposed that exosomes can direct conformational changes in Aβ forming non-toxic amyloid fibrils and promoting uptake of Aβ by microglia. The internalized Aβ is further transported by exosomes to lysosomes and degraded (Yuyama et al., 2012). In addition, they demonstrated that neuronal exosomes, but not glial exosomes, could capture Aβ (Yuyama et al., 2014, 2015). These exosomes had abundant glycosphingolipids (GSLs) essential for Aβ binding on the exosome surface. After infusion of neuron derived exosomes into brains of APP transgenic mice, Aβ and amyloid depositions decreased (Yuyama et al., 2014, 2015). These results suggest that development of engineered nanovesicles could be a profitable tool in therapy.

Another protective action against Aβ is representing by exosomal insulin-degrading enzyme (IDE). IDE is the most relevant protease that assists in the degradation of endogenous Aβ. This enzyme was found in exosomes (Bulloj et al., 2010). The proteolytically-active plasma membrane associated-IDE is led in living N2a cells (mouse NB cells) to multivesicular bodies and subsequently to exosomes. Specifically, it has been showed that hypoxia enhanced the exosome secretory rate, and increased the extracellular levels of IDE. On the other hand, it's worth noting that hypoxia promotes Aβ over-production. Thus, under hypoxic conditions IDE is overexpressed and released by exosomes in an attempt extracellular Aβ degradation.

In another work, by Tamboli et al., was demonstrated a novel pathway for the secretion of IDE via exosomes involving statins (Tamboli et al., 2010). Cellular and *in vivo* studies suggested that statins can decrease the formation of the Aβ from APP, and authors showed that statins stimulate the degradation of Aβ activating the secretion of IDE in association with exosomes (Tamboli et al., 2010). That's interesting if we consider that epidemiological studies suggested that elevated levels of cholesterol increase the risk for Alzheimer's disease (Xue-Shan et al., 2016); thus, certain studies indicated the beneficial effects of statins against Alzheimer's disease (Haag et al., 2009).

The changes in cholesterol metabolism perform an important function also in senile plaques formation and in the excessive phosphorylation of tau protein (Di Paolo and Kim, 2011; Xue-Shan et al., 2016).

Tau is a brain microtubule-associated protein that directly binds microtubule regulating its structure and function (Medina et al., 2016). The excessive phosphorylation of the tau protein is recognized as a factor which triggers formation of neurofibrillary tangles (NFT), intraneuronal inclusions that are composed of straight and paired helical filaments (PHF) (Grundke-Iqbal et al., 1986; Alonso et al., 2001). It has been proposed that these events contribute to the diffusion of neurodegeneration.

Phosphorylated tau protein was found associated with exosomes which were isolated from blood and CSF of Alzheimer's disease patients (Saman et al., 2012; Fiandaca et al., 2015). Polanco et al. showed that exosome-like EVs, isolated from the extracellular space of the mouse brain, contained significant amounts of tau, and it was differentially phosphorylated (Polanco et al., 2016). Moreover, they provided evidence that these vesicles containing tau were capable to induce tau aggregation in a threshold-dependent manner. This indicated a role for EVs in the transmission and spreading of disease. The alteration of tau protein in different cellular compartments was investigated and it has been found that tau is mainly secreted into extracellular space via exosomes (Tang et al., 2015). The translation, phosphorylation, and aggregation of tau in Alzheimer's disease brains are related to mTor (Li et al., 2005; Tang et al., 2015). mTor is involved in the intracellular tau accumulation and in its translocation as seen in Alzheimer's disease brains and cellular models (Tang et al., 2015).

Asai and colleagues demonstrated that exosomes secreted from microglia contribute to spreading tau. Indeed, the inhibition of exosomes production decreased tau propagation. By isolating exosomes from brain of PS19 mice (mice which develop tau aggregation) the authors demonstrated the presence of tau *in vivo* and these exosomes were able to transfer tau to neurons *ex vivo* (Asai et al., 2015). Besides, the microglial depletion removed exosomal transmission of tau to neurons. Moreover, the secretion of exosomes seemed to be related to ceramide synthesis, since exosomes release was sensitive to nSMase2 inhibition. In summary, they found that exosomes from microglia contribute in spreading tau pathology in brain.

At present, only few studies applied a proteomics approach to describe exosomes in Alzheimer's disease. Beside the work by Ghidoni et al. (2011), a recent work by Musunuri and collegues applied label-free shotgun mass spectrometry to profile post mortem temporal neocortex samples from Alzheimer's disease. They performed a comparison with samples from non-demented controls and other neurological disorders. After validation by antibody suspension bead arrays, they describe five representative proteins (VAMP2, TALDO, PI42A, HSP72, and CD9) and GFAP, a marker for neuroinflammation (Musunuri et al., 2016). Further works are desirable to characterize exosome and to define early biomarkers for Alzheimer's disease.

Indeed, exosomes may have also an important role as diagnostic target. It is essential to detect early diagnostic biomarker to identify who is at risk before the occurance of the neuronal loss, since most of patients do not show symptoms in the pre-clinical stages of pathology.

Exosomal tau was found in human CSF samples and it is phosphorylated at Thr-181, an accepted p-tau biomarker for Alzheimer's disease (Saman et al., 2012). In addition, the proteins co-purified with tau in exosomes, resulted linked to multiple aspects of the pathogenesis of Alzheimer's disease (Saman et al., 2014).

Fiandaca and collegues have used a combination of immunochemical and chemical appoaches to collect and enrich exosomes from small volumes of plasma or serum (Fiandaca et al., 2015). Their results demonstrated that levels of P-T181-tau, P-S396-tau, and Aβ1–42 in exosomes predict the development of Alzheimer's disease up to 10 years before clinical onset (Fiandaca et al., 2015). On the other hand, Shi and colleagues recently found that, even if tau is related to both Alzheimer and Parkinson diseases, the mechanism of tau transport is different in these two pathologies and CNS-derived plasma exosomal tau is considered more an indicator of Parkinson's disease respect to Alzheimer's disease (Shi et al., 2016).

The neural exosomic contents of transcription factors were different between controls and Alzheimer's disease patients. In particular, low exosomal levels of survival factors (i.e., LRP6, REST, and HSF1) were found in Alzheimer's disease (Goetzl et al., 2015). Also miRNAs hold great promise as diagnostic biomarkers. The expression of miRNAs and other small RNAs in plasma fraction enriched in exosomes was measured, revealing a profile of miRNA changes occurring in Alzheimer's disease (Lugli et al., 2015). Actually, as suggested by Lugli et al., the presence of 7 miRNAs was sufficient to allow highly accurate prediction of group identity in individual samples.

miRNA were also detected in exosomes extracted from CSF. Molecular changes in the brain are reflected in CSF composition, thus the CSF represents an optimal source of biomarkers for neurodegenerative diseases. The study by Gui et al. revealed a substantial difference in expression of RNA molecules in CSF exosome of Alzheimer's disease patients (Gui et al., 2015). Finally, the group of Goetzl and collaborators has recently devoted to study plasma exosomes in Alzheimer's disease. They identified plasma neuronal-derived exosomes (NDEs) and the content of synaptic proteins (synaptophysin, synaptopodin, synaptotagmins, GAP43, and neurogranin) was quantified by ELISA kits (Goetzl et al., 2016a). Levels were lower in patients with Alzheimer's disease than in cognitively normal controls. Moreover, in a different paper, the group added the investigation of astrocyte-derived exosomes (ADEs). By ELISA quantification of cargo proteins they examined the Ab42-generating system and found that γ-secretase, ADE levels of β-site amyloid precursor protein-cleaving enzyme 1, soluble amyloid precursor protein (sAPP)β, soluble Aβ42, sAPPα, glial-derived neurotrophic factor (GDNF), P-S396-tau, and P-T181-tau were significantly higher for patients, suggesting a role for activated astrocyte-neuron axis in proteinopathic dementias (Goetzl et al., 2016b). So, their effort of isolating immunochemically human plasma NDEs and ADEs, containing neuron-specific cargo, permits to characterize CNS-derived exosomes in living humans.

### Parkinson's disease

Parkinson's disease is a progressive neurodegenerative disease whose symptoms worsen over time. These include resting tremor, postural instability, bradykinesia. Whereas, the clinical and pathological features of Parkinson's disease remain to be characterized, an ascertain feature is the aggregation of a disease specific protein: α-synuclein which has dopaminergic neurotoxicity.

α-synuclein is one of the constituents of Lewy bodies and it is a presynaptic neuronal protein which binds small synaptic vesicles. Recent works have proposed that it takes part to endocytosis of synaptic vesicles. From a structural point of view, α-synuclein is formed of 140 amino acids with 3 regions. The N-terminal forms α-helix and plays a role in the binding of phospholipid vesicles, the C-terminal region has a chaperone-like function, the “non-Aβ component” is important for aggregation (Lautenschläger et al., 2017). This protein plays a central and undoubted role in the disease process, since it is the main component of abnormal protein deposits: Lewy bodies (LBs) (Vella et al., 2016).

At the beginning α-synuclein was thought to exert its pathogenic effects only intracellularly, but this concept was changed when it was detected in human plasma and CSF (El-Agnaf et al., 2003). Thus, one important characteristic of α-synuclein was found to be its inter-neuronal transmission, and exosomes have been proposed to participate in the diffusion of these proteins within the brain. Emmanouilidou et al., studying SH-SY5Y cells, demonstrated that α-synuclein is exported via exosomes and that it can impact the viability of neighboring neurons, propagating Parkinson's disease (Emmanouilidou et al., 2010). This action may be triggered by the fact that exosomes provide the catalytic environments for nucleation and acceleration of α-synuclein accumulation (Grey et al., 2015). This is in agreement with a recent study, in which exosomes were isolated from CSF of patients with Parkinson's disease. Authors found that levels of exosomal α-synuclein were related with the severity of cognitive impairment; moreover, CSF exosomes derived from Parkinson's disease induced oligomerization of α-synuclein in a dose-dependent manner (Stuendl et al., 2016).

In another study, Alvarez-Erviti et al. proved that exosomes which were emanated from α-synuclein over-expressing SH-SY5Y cells, included α-synuclein. Moreover, these exosomes could efficiently transfer α-synuclein protein to normal SH-SY5Y cells (Alvarez-Erviti et al., 2011b). Furthermore, given the evidence of lysosomal dysfunction in Parkinson's disease brains (Alvarez-Erviti et al., 2010), they inhibited lysosomal function; this led to an increase in the release of α-synuclein in exosomes togheter with an increased α-synuclein spreading to recipient cells (Alvarez-Erviti et al., 2011b).

Danzer and co-authors tried to characterize this α-synuclein associated with exosomes. Interestingly, they identified α-synuclein oligomers in exosomes and found that these exosomal α-synuclein oligomers are more toxic to neighboring cells than exosome-free α-synuclein oligomers (Danzer et al., 2012).

Other works supported the neuro-toxic role of exosomes in Parkinson's disease. Chang et al. investigated exosomes derived from mouse microglia which were induced by α-synuclein. They discovered that α-synuclein can determine an increased exosomal secretion by microglia, moreover, the activated exosomes may increase apoptosis supporting hypothesis that exosomes might mediate neurodegeneration (Chang et al., 2013).

It's worth mentioning that, even if α-synuclein is considered as an ascertain biomarker for Parkinson's disease when tested in CSF, the levels of exosomal α-synuclein in CSF were found lower in Parkinson's disease patients compared to controls. On the other hand, the levels of plasma exosomal α-synuclein were mainly higher in Parkinson's disease patients (Shi et al., 2014). Indeed, the performance of these α-synuclein species in differentiating subjects with Parkinson's disease from controls was close to that of CSF α-synuclein in terms of sensitivity and specificity. Furthermore, a significant correlation between plasma exosomal α-synuclein and disease severity was observed by Shi et al. (2014). All this data support also a role for exosomes in diagnosis.

These works suggest that preventing the α-synuclein exosomal release and uptake can be a novel approach to stop disease diffusion in Parkinson's disease.

All this evidence supports the idea that α-synuclein aggregation can be spread between neurones and this mechanism is responsible for the gradual progression of Parkinson's disease. Therefore, the decrease of α-synuclein expression is supposed to reduce this process and therefore it is an attractive approach for delaying or stopping Parkinson's disease progression, and exosomes may have a therapeutic potential (Alvarez-Erviti et al., 2011b; Cooper et al., 2014). Cooper and co-workers used exosome delivery of α-synuclein siRNA to reduce total and aggregated α-synuclein levels in mouse brains. They actually found significant decrease in intraneuronal protein aggregates, also dopaminergic neurones of the substantia nigra (Cooper et al., 2014).

Besides α-synuclein, new biomarkers are emerging. Genetic studies identified mutations in the leucine-rich repeat kinase 2 (LRRK2) gene as a putative cause of inherited Parkinson's disease (Gilks et al., 2005). The kinase domain with the most common LRRK2 mutation can direct autophosphorylation and one of the most abundant residues of autophosphorylation is Ser-1292. In a recent work, Fraser et al. measured exosome Ser(P)-1292 LRRK2 phosphorylation in urine from both idiopathic Parkinson's disease cases and controls. Ser(P)-1292 LRRK2 levels were found elevated in Parkinson's disease and correlated with features of Parkinson's disease. In particular, the authors described that these levels were connected with the severity of cognitive impairment and difficulty in accomplishing activities of daily living (Fraser et al., 2016). Therefore, Ser(P)- 1292 LRRK2 may be a useful candidate biomarker for further exploration of Parkinson's disease.

For that concerning the therapeutic potential of exosomes in Parkinson's disease, it has been further investigated by Haney et al. They developed a new delivery system, based on exosomes, for catalase, a renowned antioxidant, for the treatment of Parkinson's disease. Nanoformulated catalase was loaded into exosomes *ex vivo* which were tested in neuronal cells *in vitro* and Parkinson's disease mouse brain. Exosomal catalase showed efficient neuroprotective effects both in *in vitro* and *in vivo* models of Parkinson's disease (Haney et al., 2015).

The same group pursued the study on neuroprotective role for exosomes focusing on the glial cell-line derived neurotropic factor (GDNF) (Zhao et al., 2014). The systemic administration of GDNF-expressing macrophages was found to significantly improve neuroinflammation and neurodegeneration in Parkinson's disease mice (Biju et al., 2010). Zhao et al. discovered that exosomes have the extraordinary property of abundantly adhering and overflowing neuronal cells, and this mechanism may play a significant role in GDNF-mediated protection effects. Indeed, it facilitated the protein transfer into target neurons. Moreover, the membranotropic properties of GDNF-carrying exosomes can help GDNF binding to GFRa-1 receptors expressed on dopaminergic neurons, as affirmed by Biju et al. (2010).

At present, there are no therapies able to halt the course of Parkinson's disease, in addition, the inability of most therapeutics to cross the blood brain barrier requires the need of developing new drug delivery systems. In this perspective, exosomes have a potential as versatile strategy to treat neurodegenerative disorders because they can cross blood brain barrier.

### Multiple sclerosis

Multiple sclerosis (MS) is a chronic and inflammatory demyelinating disorder that affects the CNS. MS has risen from 2.1 million in 2008 to 2.3 million in 2013 (Browne et al., 2014). In MS, the immune system attacks myelin and results in axonal degeneration in the brain and spinal cord, leading to severe neurological disability. A natural repairing process consists of the spontaneous remyelination which is mediated by recruitment of oligodendrocyte precursor cells (OPCs) to lesions, and their following differentiation into myelinating oligodendrocytes (Kuhlmann et al., 2008). However, this ability progressively declines making worse the disease progression (Pusic et al., 2014a).

Scolding et al. were the first to describe the presence of oligodendroglial vesicles in the CSF of patients with MS (Scolding et al., 1989) opening to the study of exosomal involvement in MS.

Verderio et al. identified vesicles shed by myeloid cells as therapeutic targets and markers for MS. In particular, they detected increased myeloid vesicles concentration in CSF from mice and MS patients. Their data suggested that myeloid EVs may enhance excitatory transmission (Antonucci et al., 2012) interacting with neurons plasma membrane and propagate neuroinflammation (Verderio et al., 2012). Moreover, FTY720, the first approved oral MS drug, was shown to considerably reduce EVs in CSF from mice model of MS. The work by Minagar et al. explored the aspect of endothelial cell dysfunction, which has been proposed to be related with MS. They found in plasma from MS patients higher level of EVs released from endothelial cells respect to healthy subjects. These vesicles contained CD31 elevation from patients in exacerbation whereas CD51 levels were high both in remission and exacerbation. This result suggested that CD31 could be related to acute disease, while CD51 seems to reflect chronic injury to endothelium (Minagar et al., 2001).

The same group further investigated the level of CD31 in endothelial EVs from patients with relapsing-remitting MS, prior and after starting therapy with IFN-beta1a. The levels of CD31 were significantly decreased after 24–52 weeks from the initiation of therapy suggesting that IFN-beta1a may have a normalizing effect in MS on cerebral endothelial cells (Sheremata et al., 2006). Furthermore, they studied EVs derived from platelets. They started from the observation of platelet activation in MS. They found a significant increase of EVs from platelets in MS patients along with the elevation of CD62p expression, which is a marker of platelets activation (Sheremata et al., 2008). P-selectin from platelet-derived EVs can bind PSGL-1 and PECAM-1 from lymphocytes supporting their binding to the endothelium through the promotion of integrins expression (Sheremata et al., 2008). A study by Sáenz-Cuesta and colleagues focused on vesicles derived from 3 different cells (i.e., leukocytes, monocytes, platelets) from MS patients and controls. The platelet-derived EVs were more abundant in untreated MS patients respect with controls (Sáenz-Cuesta et al., 2014). Moreover, the count of EVs from the 3 subtypes was higher in relapsing-remitting patients whereas secondary progressive patients had levels similar to controls. Therefore, the authors proposed that the spreading of EVs may increase during inflammation and reduce to baseline during chronic degeneration (Sáenz-Cuesta et al., 2014).

Since myelination in the CNS is regulated by miRNA (Dugas et al., 2010; Zhao et al., 2010), some studies focused on miRNAs present in exosomes. Pusic et al. found that serum exosomes which were derived from young animals enhance CNS myelination partially by delivering miR-219 (Pusic and Kraig, 2014). Interestingly, they obtained the same result with exosomes derived from environmental enrichment (EE) rats. EE is defined by the authors as volitionally increased physical, social, and intellectual activity that ameliorates production of myelin at all ages (Pusic et al., 2016). Indeed, they showed that these exosomes increased OPCs and their differentiation, and also ameliorated remyelination after lysolecithin-induced demyelination in brain slice culture (Pusic and Kraig, 2014) and reduced oxidative stress (Pusic et al., 2016). This effect is mediated by the enrichment in exosomes of miR-219, which reduces the expression of inhibitory regulators of differentiation, supporting the production of myelinating oligodendrocyte. This effect was found by Pusic et al. also in *in vivo* experiments, since nasal administration of young serum-derived exosomes significantly increased myelin in aged rats (Pusic and Kraig, 2014).

This work has been carried on by exploring the use of dendritic cells as an exogenous source of pro-myelinating exosomes; it was found that stimulation with IFNγ induced production of exosomes containing high levels of miR-219 (Pusic et al., 2014b). When applied to slice cultures, these exosomes increased the levels of myelin basic protein, which resulted in the production of structurally normal and thicker myelin sheaths (Pusic et al., 2014b). Thus, these results suggested that peripheral circulating cells can produce exosomes that may be an effective treatment for remyelination.

The presence of miRNA in MS exosomes has been further investigated by Giovannelli and co-workers. Two miRNA, named miRNA-J1-3p and miRNA-J1-5p, have been identified in exosomes obtained from plasma and urine (Giovannelli et al., 2015). These are two miRNAs from Polyomavirus JC (JCPyV). JCPyV reactivation and development of progressive multifocal leukoencephalopathy (PML) is a health concern in MS patients under natalizumab therapy. Natalizumab is the first humanized monoclonal antibody indicated in the treatment of relapsing-remitting MS. Therefore, they thought to investigate the presence of these two miRNAs in samples from MS patients before and during natalizumab therapy and in healthy donours. Specifically, the increased JCPyV miRNA expression in samples from MS patients under natalizumab therapy was consistent with the coinciding high JCPyV-DNA positivity (Giovannelli et al., 2015). Taken together, the data suggested that monitoring alterations in the expression of the JCPyV miRNA can be helpful to clarify the early virus activation mechanism, but also can have a clinical significance in identifying patients at risk of PML.

### Amyotrophic lateral sclerosis

Amyotrophic lateral sclerosis (ALS) is a fatal, progressive neurodegenerative disease that affects the corticospinal tract, leading to motor neuron death in the cortex, brainstem, and spinal cord. It is a rare disease, with a mean incidence rate of 2.8/100,000 in Europe and 1.8/100,000 in North America, and a mean prevalence rate of 5.40/100,000 in Europe and 3.40/100,000 in North America (Chiò et al., 2013; Bozzoni et al., 2016). Median survival is 2–4 years from onset (del Aguila et al., 2003). The disease is characterized by progressive muscle atrophy, increased fatigue, and problems with swallowing, which usually leads to respiratory failure and death (Rowland, 1994; Chiò et al., 2013). The majority of cases are sporadic (SALS), while 5–10% is familial (FALS); the role of genetic and environmental factors in sporadic cases is still unknown.

Up to 20% of FALS cases contain a mutation in the gene coding Cu/Zn superoxide dismutase 1 (SOD1), and more than 100 mutations in the protein SOD1 have been identified (Kaur et al., 2016). SOD1 protects cells from superoxide radicals by converting them into molecular oxygen and hydrogen peroxide, and it is typically considered a cytosolic enzyme. But it has been found in the extracellular environment, under physiological and pathological conditions, secreted by astrocytes, fibroblasts, spinal cord cultures, and motor neuron-like NSC-34 cells (Mondola et al., 1996; Lafon-Cazal et al., 2003; Turner et al., 2005; Urushitani et al., 2006; Basso et al., 2013). Moreover, SOD1 has been discovered in CSF of ALS patients (Jacobsson et al., 2001).

The first which observed SOD1 associated with exosomes in ALS were Gomes et al. In their work, they have shown that wild type (SOD1wt) and mutant SOD1 (SOD1G93A) were in the supernatant medium from NSC-34 ALS cell model stably expressing hSOD1wt/G93A. This protein was associated with exosomes (Gomes et al., 2007). Mutant hSOD1 was found in exosomes, but it seemed to be less incorporated respect to hSOD1wt, therefore authors supposed that it could be caught inside the cell, taking part to the damaging effect. Moreover, the presence of SOD1G93A in the extracellular media through exosomes, even if in a little amount, could trigger inflammation. Hence Gomes and collaborators proposed a role for extracellular mutant SOD1 in the pathogenesis of ALS (Gomes et al., 2007).

The detrimental effect of mutant SOD1 was supported by Basso and co-workers in a study on mice expressing mutant SOD1, which are the best characterized mouse model of FALS. They showed that astrocytes with up-regulation of SOD1G93A were characterized by a bigger release of exosomes, which transfer mutant SOD1 to spinal neurons inducing selective motor neuron death (Basso et al., 2013). The work suggested that astrocytes, and other brain cells, use exosome release as a means of clearance for misfolded and likely toxic proteins, such as mutant SOD1. By this way, astrocytes could limit the formation of intracellular aggregates. On the other hand, this exosome release can exert a toxic effect on neighboring motor neurons cells and is thought as a way for disease spreading in neurodegenerative disorders (Basso et al., 2013).

Subsequently, even if the possibility that SOD1 misfolding derived from SALS cannot be ruled out, it has also been proposed that the propagation of misfolded SOD1 could sustain all types of ALS, included the sporadic form. Indeed, Grad et al. reported that misfolded hSOD1wt can be released via exosomes, and uptaken in neuronal cells (Grad et al., 2014). Misfolded hSOD1wt can support the intercellular diffusion of misfolding *in vitro* and is detected in the spinal cord of all ALS patients tested, representing about 4% of total SOD1. Hence, their data indicate that SOD1 participates in propagated misfolding, indicating a common pathogenic mechanism between FALS and SALS (Grad et al., 2014). This was supported also by previous evidence that mutant SOD1 confers its misfolded aggregated phenotype on soluble mutant SOD1, and this does not depend from contacts among cells but it is related on the extracellular release of aggregates (Münch et al., 2011).

Another area of interest in studying ALS are adipose-derived stromal cells (ASCs). ASCs have a potential therapeutic application, related to their easy availability and ability to move toward damaged tissues, contributing to their repair (Bonafede et al., 2016). In addition, Marconi et al. have proposed that *in vivo* administration of ASCs ameliorates disease in murine ALS, promoting neuro-protection and neuro-regeneration (Marconi et al., 2013). In the work by Bonafede et al., exosomes isolated from ASCs were used to test their potential neuroprotective effect on an *in vitro* model of ALS motoneurons against H_2_O_2_-induced damage (Bonafede et al., 2016). Their findings in the NSC-34 model of motoneurons showed that exosomes could prevent the oxidative damage; indeed, exosomes exerted a neuroprotective effect on cell viability increasing their resistance and survival to oxidative damage. The authors believed that the beneficial effect of ASCs exosomes could be caused by the secretion of miRNAs which have a protective role via apoptosis-inhibiting pathway, cell cycle progression and proliferation (Bonafede et al., 2016). More recently, exosomes from adipose-derived stem cells (ADSC-exo) were proposed as potential candidates of therapeutic effects (Lee et al., 2016a). The authors suggested that ADSC-exo modulate cellular phenotypes of ALS together with SOD1 aggregation and mitochondrial dysfunction, and can represent a putative therapeutic target for ALS. In particular, beneficial effects of ADSC-exo may be derived from restoration of mitochondrial functions (Lee et al., 2016a).

In view of all these findings, a novel therapeutic approach could consider to target the exosomes as means able to halt the course of ALS.

### Huntington's disease

Huntington's disease (HD) is a neurodegenerative disorder which is characterized by the decline of cognitive, motor and behavioral state. The age of onset is typically on the 4th decade but it varies from 20 to 65 years; HD inexorably advances over 20 years, and there is no treatment to delay neurodegeneration, therefore it is basically fatal. HD is an autosomal dominant disease resulting from a mutation in the gene coding huntingtin (HTT) on chromosome 4. This mutation consists in the expansion of trinucleotide repeat CAG (Apolinário et al., 2017; Wyant et al., 2017). This mutant HTT contains polyglutamine (polyQ) repeat and it has been proposed that both the expanded repeat RNA and the polyQ repeat could mediate the neurotoxicity in HD. Indeed, the mutant HTT can homo/heterodimerize forming protein aggregates and fibrils which determine neurodegeneration (DiFiglia et al., 1997; Zhang et al., 2016). Neurodegeneration could propagate in SNC through EVs.

Zhang and colleagues evaluated the transmission to striatal neural cells *in vitro* of EVs with polyQ HTT protein, expanded repeat CAG RNA or normal protein. Indeed, they discovered that EVs integrated both expanded repeat RNA and polyQ protein. Nevertheless, no toxicity emerged after exposure to vesicles for 72 and 96 h. Authors supposed it could be related to a short experimental interval if compared with the exposure period *in vivo*. However, they believe that it's worth to assess the presence in EVs from biological fluids from HD patients of polyQ HTT protein and expanded repeat CAG RNA, both as biomarker and target for treatment (Zhang et al., 2016). On the other hand, the putative therapeutic efficacy of exosomes has been exploited.

For example, a work by Didiot et al. inquired into the feasibility of using exosomes for treatment delivery. For this study they tested hydrophobically modified siRNAs (hsiRNAs) which are siRNA modified for improving cellular internalization. Indeed, siRNA belong to a new class of therapeutics which target RNA to avoid expression of the protein related to the disease. However, the clinical treatment of neurodegenerative disease with siRNA is hindered by improper CNS diffusion. Instead, they observed that co-incubation of hsiRNAs with exosomes was an effective method for loading vesicles with specific oligonucleotides. The unilateral infusion of exosomes loaded with hsiRNA into mouse striatum, determined a bilateral oligonucleotide distribution and a statistically significant bilateral silencing of up to 35% of HTT mRNA. These results provide a noteworthy advance for the development of new treatments of HD and other neurodegenerative diseases (Didiot et al., 2016).

The group of Lee et al., studied the therapeutic efficacy in HD of peculiar exosomes that they studied also in ALS: the ADSC-exo (Lee et al., 2016b). They used *in vitro* HD model for examining the effects of ADSC-exo on modulation of apoptosis and mitochondrial function. They actually showed a decrease in mutant HTT accumulation, in apoptosis and a protective role in mitochondrial function. Therefore, they confirmed the therapeutic effect of ADSC-exo in HD's treatment (Lee et al., 2016b).

The role of miRNA has emerged also in HD. In particular, miRNA-124 was found repressed in HD (Johnson et al., 2008). The decrease of miRNA-124 raises the expression of REST, its target gene and, as a consequence, there is the inhibition of genes, such as brain-derived neurotrophic factor. Moreover, miRNA-124 promotes neurogenesis therefore its delivery can be a possible way for inducing neural regeneration. Lee and colleagues generated exosomes containing miRNA-124 which, when delivered to the striatum, lowered REST expression. The treatment with exosomes containing miRNA-124 had a limited therapeutic efficacy, however, this study represented a first step for the use of exosome in delivering miRNA to the brain (Lee et al., 2017).

### Prion diseases

Transmissible spongiform encephalopathies (TSEs), also called prion diseases, are a class of rare, transmissible neurodegenerative diseases which include Fatal familial insomnia (FFI), Creutzfeldt-Jakob disease (CJD), Gerstmann-SträusslerScheinker disease (GSS) and Kuru and can appear as genetic, infectious or sporadic disorders. These diseases are characterized by abnormal accumulation in the CNS of pathologic prion protein (PrP^sc^), the misfolded form of the normal cellular prion protein (PrP^c^), which causes grave neuronal damages leading to dementia (Prusiner, 1996). PrP^c^ is a glycosylphosphatidylinositol (GPI)-linked extracellular membrane protein, present in both neuronal and glial cells. Its biological function is not clearly understood, and it has been proposed that can take part to many cellular activities in CNS, such as synapse formation and neurite growth (Harischandra et al., 2014).

Over the last decade, it has been discussed the role of EVs as carriers of PrP^sc^ within the CNS. Actually, prion agent seems to be released from the cell in association with exosomes contributing to spread neurodegeneration (Fevrier et al., 2004; Vella et al., 2007; Alais et al., 2008).

Saá and collaborators isolated EVs from plasma collected from mice infected by mouse-adapted variant of CJD (Mo-vCJD) and from the medium of cell cultures which were infected by Mo-vCJD or Fukuoka-1 (a mouse-adapted isolate of a patient with GSS). They discovered the presence of PrP^sc^ in vesicles and with the biochemical characterization they demonstrated also the presence of Hsp70, an exosomal marker. Therefore, for the first time, a specific relation of a fraction of blood-circulating PrPsc with exosomes was proved. Since exosomes can cross the blood-brain barrier, this result suggested the role of these vesicles in spreading prion disease through blood (Saá et al., 2014) albeit they did not give direct evidence. Therefore, the same group carried on this research (Cervenakova et al., 2016). They isolated vesicles from plasma of mice infected by Fukuoka-1, and subjected samples to multiple rounds of protein misfolding cyclic amplification (PMCA) demonstrating that they contain PrP^sc^. Moreover, they reported that these EVs where able to propagate the infection if injected into Tga20 mice, transgenic mice which over-express mouse PrP^c^, corroborating the proposal that transmission of prion occurs via exosomes. Guo and collaborators addressed the issue about the process of PrP packaging into exosomes. They investigated the putative role of neutral sphingomyelinase (nSMase) pathway in exosome formation and inclusion of PrP (Guo et al., 2015). Through the inhibition of nSMase pathway with GW4869 (a chemical inhibitor) they demonstrated its role in exosome biogenesis and PrP packaging. While, with the use of nSMase1 and nSMase2 knockdown in mouse neurons, they proved that packaging of PrP^c^, but not PrP^sc^, is dependent on nSMase2. Besides, nSMase1 has more nSMase-activity in cells infected by prion respect to uninfected cells. Thus, the authors contributed to understanding pathways involved in transmission of PrP via exosome. The same group worked for further exploring the spread of prions through EVs. They applied the transwell assay in which two populations of cells (infected and non-infected with prions) were co-cultured separated by a semipermeable membrane that avoided their direct contact (Guo et al., 2016). By this way, they demonstrated that infection took place thanks to exosomes. Moreover, when chemical compounds which increase (monensin) or decrease (GW4869) the release of exosomes were used, they observed a linear connection with increase or decrease in prions transmission. In addition, monensin can modify the conformational stability of PrP^c^ causing the increase of PrP^sc^. Some *in vivo* studies could give strength to these results.

As seen in the other described neurodegenerative diseases, the presence of miRNA was evaluated also in prion diseases. Some miRNA were found deregulated in brain tissues from mice infected with prion (Saba et al., 2008) and the work by Bellingham et al. dealt with the miRNA signature in exosomes emitted by neuronal cells infected by prion (Bellingham et al., 2012). By a deep sequencing of small RNA, the authors found a characteristic signature of miRNAs (increased let-7i, let-7b, miR-21, miR-128a, miR-29b, miR-222, miR-424, and miR-342-3p levels with decreased miR-146a levels) in exosomes emitted by prion infected neuronal cells respect to exosomes from non-infected cells. This may have a relevant importance in diagnosis but also in defining the role of exosomes during prion propagation.

## Conclusions

In summary, studies on the role of exosomes in neurological disorders are continuously expanding. Vesicles have been proposed as promising biomarkers as well as suitable therapeutic agents.

In GB the immune-modulating properties of exosome have emerged related to their cargo in HSPs capable of driving antitumor immunity, and the presence of canonical HSPs was confirmed also in MB. On the other side, in NB, vesicles seem to have a main role in supporting cancer progression, as demonstrated by the presence of proteins modulating the tumor microenvironment but also by exosomal miRNAs (e.g., miR-21, miR-155) which affect NB resistance to chemotherapy. This is in line with evidence that also the presence of specific miRNAs (miR-181c, miR-122, and miR-19a) in exosomes has been demonstrated to promote brain metastasis. It is worth mentioning that, beside EVs, cell-free DNA (cfDNA), short DNA fragments of 70–200 base pair, have been proposed to be shed by cancer cells. CSF from patients with brain and spinal cord tumors were analyzed searching for tumor DNA (Wang et al., 2015). However, even if circulating DNA has a promising role in diagnosis, tumor-derived DNA represents a small part of total cfDNA, therefore the specific detection of DNA alteration is a critical aspect in cfDNA analysis which requires advanced technologies. The most promising techniques have been reviewed, and molecular barcoding appears as the suitable method for overcoming limitation of classical PCR and sequencing (Volik et al., 2016).

Beside brain cancers, in the last years we assist to a remarkable increase in publications in the field of neurodegenerative diseases and EVs. This helps in providing new insights into their role and physiology even if their function in neurodegenerative diseases is yet to be totally understood. Both in Alzheimer's and Parkinson's disease, exosomes can propagate the proliferation of misfolded proteins. In Alzheimer's disease proteins associated with exosomes, such as flotillins and Alix, are enriched in the amyloid plaques suggesting that exosome-associated Aβ can take part to plaque formation. Furthermore, members of the secretase were identified associated with exosomes, corroborating the fact that cleavage of APP may occur within these vesicles. Similarly, it has been demonstrated that α-synuclein is exported via exosomes propagating Parkinson's disease. Exosomes supply the catalytic environments for nucleation, accelerating α-synuclein aggregation. In the same direction are oriented studies on SZ, even if it is not a typical neurodegenerative disease. Indeed, it has been reported that dysbindin-1B has a tendency to aggregate in primary cortical neurons in SZ, and, once formed, the toxic aggregates are packed and propagated by exosomes. Mounting evidence suggests the contribution of EVs in spreading neurodegeneration also in prion diseases and HD.

But there is also evidence to hint that EVs can have a protective role in neurodegeneration. Cystatin C, which has a neuroprotective role in Alzheimer's disease, was found secreted in association with exosomes. Another protective action against Aβ is representing by IDE that assists in the degradation of endogenous Aβ and it was found in exosomes. In Parkinson's disease the therapeutic potential of exosomes has emerged. Indeed, exosomal catalase supplied a significant neuroprotective effect both *in vitro* and *in vivo* models. Moreover, the administration of GDNF-expressing macrophages, ameliorated neuroinflammation and neurodegeneration in Parkinson's disease mice. In addition, in ALS has been suggested that brain cells, including astrocytes, use exosome release as a means of clearance for misfolded and potentially toxic proteins, such as mutant SOD1. Some advancements have been made also in the study of MS where it has been found that the enrichment in exosomes of miR-219 improve CNS myelination.

Studies on EVs have also helped in defining new biomarkers. The levels of P-T181-tau, P-S396-tau, and Aβ1–42 in exosomes predict the development of Alzheimer's disease up to 10 years before clinical onset. Moreover, low exosomal levels of LRP6, REST, HSF1 were found in Alzheimer's disease. But also levels of synaptopodin, synaptophysin, synaptotagmins, neurogranin and GAP43 were lower in patients with Alzheimer's disease than in cognitively normal controls. Finally, a profile of miRNA changes in plasma fraction enriched in exosomes has been revealed and miRNA were also detected in exosomes extracted from CSF. The role of miRNA has emerged also in HD. In particular, miRNA-124 was found repressed in HD. A characteristic signature of miRNA have been found also in exosomes emitted by prion-infected neuronal cells. Concerning Parkinson's disease, Ser(P)- 1292 LRRK2 may be a useful candidate biomarker since its levels were found elevated and correlated with features of Parkinson's disease.

**Figure 1:**
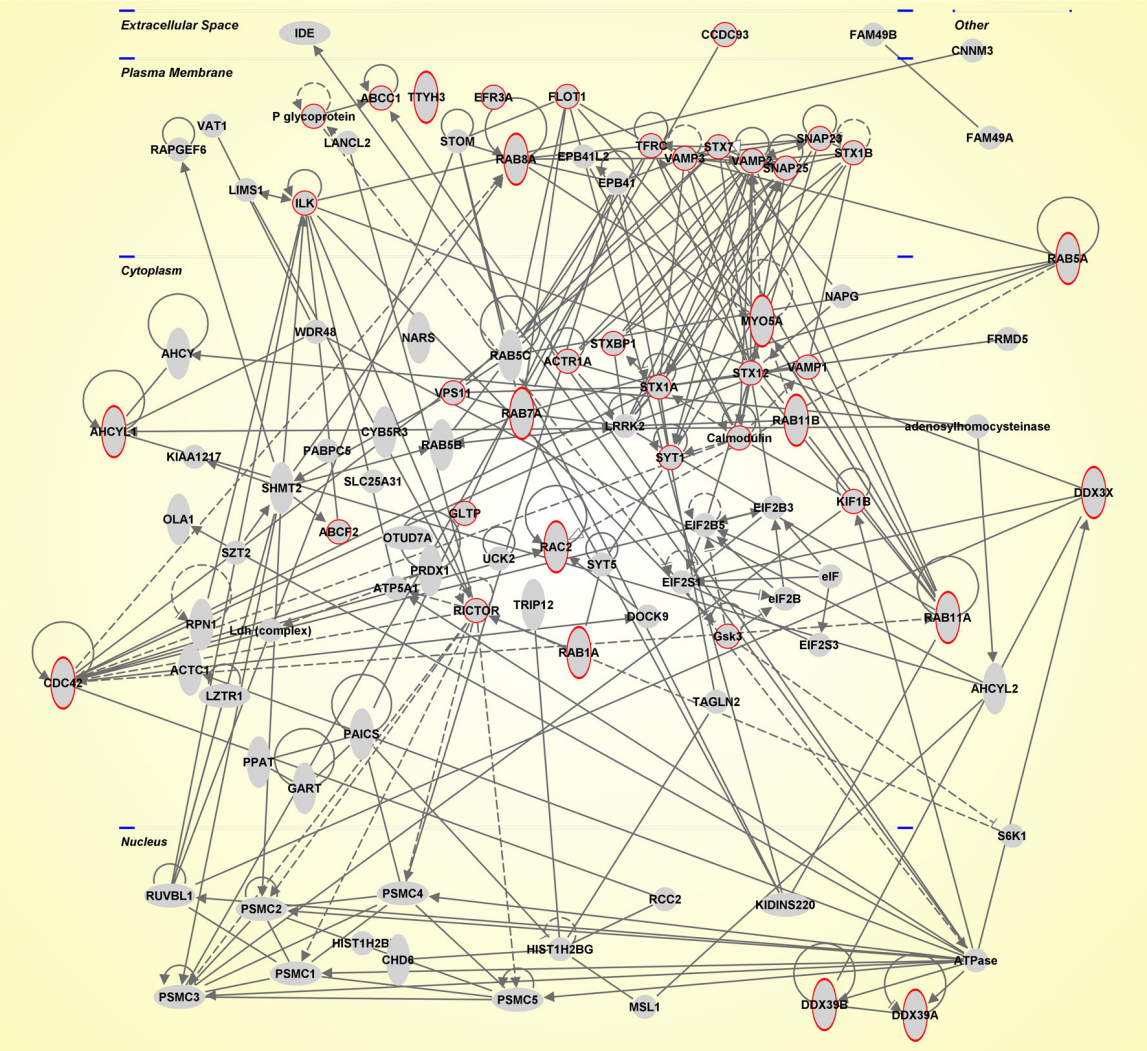
Ingenuity Pathway Analysis. Network suggested by QIAGEN's Ingenuity Pathway Analysis (IPA, QIAGEN Redwood City, USA. www.qiagen.com/ingenuity) for the molecules identified in EVs, by focusing on the SNC, that were present in databases. The network is depicted with the subcellular layout. Red circles highlight the top function linked with the network: “secretory pathway” (*p*-value 3.4e^−14^).

In conclusion, as emerged by this review, there is a huge amount of data on EVs which is exponentially increasing (Table 1). In this perspective, the scientific community contributed to the development of databases. Exocharta (http://www.exocarta.org/), and Vesiclepedia (http://www.microvesicles.org/) are two of these databases with a compendium of lipids, RNA and proteins identified in different classes of EVs. Here, we extracted molecules identified in EVs by focusing on the SNC (Table S1). All these molecules were functionally analyzed by using QIAGEN's Ingenuity Pathway Analysis (Ingenuity® System Inc., QIAGEN Redwood City, CA, USA) and thus predominant canonical pathways and interaction networks were obtained. Indeed, IPA includes the Core Analysis function, which helps to interpret the data in the context of biological networks. The major categories of network-associated functions included “Cellular Assembly and Organization, Cellular Function and Maintenance” (score value 45, Figure 1). Moreover, it is noteworthy that, as expected, the top function linked with the network was the “Molecular Transport” and its sub-category “secretory pathway” (*p*-value 3.4e^−14^) that is associated with the red circled proteins (Figure 1).

**Table 1:** Molecules present in vesicles in different biological samples.

**Biological sample**	**Biological molecules**	**References**	**Biological sample**	**Biological molecules**	**References**
**BRAIN CANCERS**					
***Glioblastoma***			***Neuroblastoma***		
Brain tumor cell lines	HSPs	Graner et al., 2007	NB cell lines	Oncogenic miRNAs	Haug et al., 2015
•Brain tumor cell lines •Serum	EGFR, EGFRvIII, TGF-beta	Graner et al., 2009	•NB cell lines •Monocytes	miRNA (miR-21 and miR-155)	Challagundla et al., 2015
Glioblastoma cell line	Antigens	Harshyne et al., 2015	NB cell lines	Proteins (e.g. fibronectin and clathrin)	Marimpietri et al., 2013
Glioma cells	CRYAB	Kore and Abraham, 2014	***Medulloblastoma***		
Serum	Immunoglobulin	Harshyne et al., 2016	MB cell lines	Proteins (e.g., G3P, ACTB, ANXA2)	Bisaro et al., 2015
•Glioblastoma cell lines •Plasma •CSF	miRNA (e.g., miR-21)	Akers et al., 2015	•MB cell lines •Serum	Proteins (e.g., HSP, ERBB2, tetraspanin)	Epple et al., 2012
Blood	mRNA (EGFR/EGFRvIII)	Shao et al., 2015	***Metastatic Brain Tumors***		
Plasma	Proteins, mRNA (immunological markers)	Muller et al., 2015	•Breast cancer •Brain astrocytes	miRNA-122	Fong et al., 2015
Serum	miRNA (miR-320 and miR-574-3p)	Manterola et al., 2014	Serum	miRNA-122	Wu et al., 2012
CSF	miRNA (e.g., miR-21)	Akers et al., 2013	Tumor models	Proteins (integrins)	Hoshino et al., 2015
Serum	Proteins, mRNA (EGFRvIII)	Skog et al., 2008	Astrocyte	miRNA	Zhang et al., 2015
GBM cell lines	Proteins (e.g., annexin A1, actin-related protein 3, integrin-β1)	Mallawaaratchy et al., 2017	•Breast cancer cells •Brain metastasis	miRNA-181c	Tominaga et al., 2015
**CHRONIC NEURODEGENERATIVE DISEASES**					
***Alzheimer's disease***			***Parkinson's disease***		
Neuroblastoma cells HeLa cells	Beta-amyloid peptides	Rajendran et al., 2006	SH-SY5Y cells	α-synuclein	Emmanouilidou et al., 2010
CHO-APP_695_ cells	APP metabolites	Sharples et al., 2008	CSF	α-synuclein	Stuendl et al., 2016
Brains of Tg2576 mice	APP, CTFs–APP, Aβ	Perez-Gonzalez et al., 2012	SH-SY5Y cells	α-synuclein	Alvarez-Erviti et al., 2011b
Astrocytes	Ceramide, PAR-4	Wang et al., 2012	Neuroglioma cells	α-synuclein oligomers	Danzer et al., 2012
Mouse primary neurons	Cystatin C	Ghidoni et al., 2011	BV-2 cells	MHC class II molecules, TNF-α	Chang et al., 2013
•N2a cells •CSF	Aβ oligomers	An et al., 2013	•CSF •Plasma	α-synuclein	Shi et al., 2014
Dendritic cells	siRNA targeted to BACE1	Alvarez-Erviti et al., 2011a	Murine dendritic cells	siRNA	Cooper et al., 2014
Brain mice	Glycosphingolipids	Yuyama et al., 2014	Urine	LRRK2	Gilks et al., 2005
N2a cells	Insulin-degrading enzyme	Bulloj et al., 2010	***Multiple sclerosis***		
CSF	Phosphorylated tau	Saman et al., 2012	Serum	miRNA-219	Pusic and Kraig, 2014
Blood	Phosphorylated tau	Fiandaca et al., 2015	Serum	miRNA-219	Pusic et al., 2016
Mouse brain	Phosphorylated tau	Polanco et al., 2016	Dendritic cells	miRNA-219	Pusic et al., 2014b
CSF	Phosphorylated tau	Saman et al., 2012	•Plasma •Urine	miRNA-J1-3p miRNA-J1-5p	Giovannelli et al., 2015
•Plasma •Serum	P-S396-tau, P-T181-tau, Aβ1–42	Fiandaca et al., 2015	Plasma	CD31, CD51	Minagar et al., 2001
Post mortem temporal neocortex	Proteins	Musunuri et al., 2016	Plasma	CD31	Sheremata et al., 2006
Plasma	Survival factors (LRP6, REST, HSF1)	Goetzl et al., 2015	Platelet	P-selectin	Sheremata et al., 2008
CSF	miRNA	Gui et al., 2015	***Amyotrophic lateral sclerosis***		
Plasma	miRNA (e.g., miR-342-3p, 342-5p,141-3p)	Lugli et al., 2015	NSC-34 cells	SOD1	Gomes et al., 2007
Plasma neuronal-derived exosomes	Synaptic proteins	Goetzl et al., 2016a	Astrocyte	SOD1	Basso et al., 2013
Astrocyte-derived exosomes	Cargo proteins	Goetzl et al., 2016b	Adipose-derived stromal cells	Protective miRNAs	Bonafede et al., 2016
CSF	Lipid (e.g., sphingomyelin, cholesterol, ceramide)	Joshi et al., 2014	NSC-34 cells	SOD1	Grad et al., 2014
Brain PS19 mice	Tau	Asai et al., 2015	Adipose-derived stem cells	SOD1	Lee et al., 2016a
***Prion diseases***			***Huntington's disease***		
Mice plasma	PrP^sc^, Hsp70	Saá et al., 2014	Striatal neural cells	PolyQ protein repeat CAG RNA	Zhang et al., 2016
Prion infected neuronal cells	miRNA (e.g., miR-21, miR-128a, miR-29b)	Bellingham et al., 2012	HEK 293 cells	miRNA-124	Lee et al., 2017

## Concluding remarks

As evidenced by a plethora of works, the studies on the role of exosomes in neurological disorders are expanding. Vesicles have been proposed as promising biomarkers as well as suitable therapeutic agents. However, there are some limitations that must be passed before their widespread clinical use. First of all, the technical challenge in the isolation of exosomes. There are many different methods in literature, for isolation and characterization of vesicles, while there is a need of more standardized conditions for obtaining reproducibility and really purified exosomes. Moreover, the characterization of post-translational modifications of exosomal proteins is lacking. To define post-translational modifications in exosomes, could allow a better understanding of their physiology and open new ways for drugs targets. Finally, extensive clinical trials, could help the translational aspect in studying exosomes.

A deeper analysis of these aspects may lead to the concrete use of exosomes for diagnosis and as novel therapy approach in neurological diseases.

## Author contributions

FC and GP organized and revised the manuscript. FC, AU, and GP equally contributed to the writing of this review. All authors approved the final manuscript.

### Conflict of interest statement

The authors declare that the research was conducted in the absence of any commercial or financial relationships that could be construed as a potential conflict of interest.
